# Measles

**Published:** 1889-12

**Authors:** 


### MEASLES.

Measles commence with all of the ordinary indications of a slight cold, such as running at the nose, red, watery eyes, and a cough. About the fourth day after the commencement of the attack, the rash begins on the face and extends over the body and limbs. By the seventh day the rash begins to fade and the fever declines. Treatment:—Begin with a dose of citrate of magnesia to open the bowels, and follow with ipecacuanha. For a drink, use flaxseed and lemonade. If there is much cough, use syrup of squills.

